# Intercostal Neuralgia Successfully Managed With Peripheral Nerve Stimulation

**DOI:** 10.7759/cureus.71964

**Published:** 2024-10-20

**Authors:** David M Gallacher, Paul Gastelum, Sungkook A Park

**Affiliations:** 1 Physical Medicine and Rehabilitation, University of Colorado, Aurora, USA; 2 Pain Management, Desert Orthopaedic Center, Las Vegas, USA

**Keywords:** chronic pain, complex regional pain syndrome, crps, intercostal neuralgia, neuromodulation, pain management, pain medicine, peripheral nerve stimulation, pns

## Abstract

Intercostal neuralgia is characterized by neuropathic pain along the distribution of the intercostal nerve, which can cause debilitating pain and interfere with daily activities. The literature is extremely limited in assessing the use of neuromodulation to treat trauma-induced intercostal neuralgia. This case reports a 40-year-old patient who presented with decades of refractory, long-standing thoracic pain. The pain ranged from a four out of 10 to a nine out of 10 on the numeric rating scale (NRS). The patient failed pharmacologic therapy, physical therapy, chiropractic care, injection therapy, transcutaneous electrical nerve stimulation (TENS), and spinal cord stimulation (SCS). The patient underwent a 60-day peripheral nerve stimulation (PNS) trial, which temporarily relieved the pain until it was explanted. The patient subsequently underwent placement of a permanent PNS implant, which provided between 80-100% daily pain relief at a six-month follow-up. At the two-year follow-up, the patient continued to experience sustained pain relief, had weaned from opioid medications, and returned to all desired daily activities. This case suggests that minimally invasive PNS can effectively manage pain for patients with intercostal neuralgia who fail conservative measures. Further, to our knowledge, this two-year follow-up is the longest-reported outcome in the literature for using PNS for intercostal neuralgia after traumatic injury.

## Introduction

Intercostal neuralgia is characterized by neuropathic pain along the distribution of the intercostal nerves, resulting in significant thoracic pain and discomfort to the chest, abdomen, and flank [1]. Neuropathic pain is a direct consequence of a lesion or disease of the somatosensory system and can be described as burning, stabbing, sharpness, numbness, or tingling [1,2]. Similar to most pain conditions, neuropathic pain can significantly limit patients’ daily activities. 

The most described use of neuromodulation for intercostal neuralgia is in post-herpetic neuralgia [2-5], a complication of the herpes zoster virus (HZV) infection, with an estimated prevalence of 3.2 cases per 1,000 person-years [6]. Additional causes include nerve injuries secondary to fractures and medical procedures (e.g., thoracotomy), which have previously been reported in the literature to cause intercostal neuralgia [7]. 

The treatment strategies for intercostal neuralgia include physical therapy, nonsteroidal anti-inflammatory drugs (NSAIDs), serotonin-norepinephrine reuptake inhibitors (SNRIs), tricyclic antidepressants (TCAs), anticonvulsants, and opioids. Newer modalities have included neurectomy, radiofrequency ablation, and spinal cord stimulation (SCS) [3,8-10]. However, the treatment of refractory intercostal neuralgia still presents a challenge for some patients. 

Peripheral nerve stimulation (PNS) represents a therapeutic option to treat chronic neuropathic pain [11]. PNS involves the application of electrical impulses to the peripheral nerves through a small, implanted generator connected to electrodes near the targeted nerve. In this case, we present the clinical use of PNS to manage intercostal neuralgia using high-frequency neuromodulation.

## Case presentation

A 40-year-old male patient presented to the pain medicine clinic with a long-standing history of left-sided mid-thoracic pain. The patient reported the pain began during adolescence following a hyperextension injury while playing sports. The pain localized to the mid-thoracic spine with radiation to the left chest wall. The intensity of the pain was eight out of 10 on average and was exacerbated with deep breathing and sleeping on his left side. The patient tried many conservative and interventional treatment modalities over the years. Failed measures included NSAIDs, lidocaine patches, gabapentin, muscle relaxers, opioids, steroid injections, transcutaneous electrical nerve stimulation (TENS), thoracic medial branch blocks, and spinal cord stimulation (SCS). The patient also failed to have improvements in the symptoms with non-pharmacologic interventions, including physical therapy and chiropractic care.

Upon presentation to the pain management clinic, the patient underwent a comprehensive history and physical exam. Further diagnostic workup was ordered to include electromyography (EMG), which failed to show notable axonal neuropathy of the intercostal nerves or posterior rami at the midthoracic spine. Further imaging via magnetic resonance imaging (MRI) showed multilevel degenerative disc disease (DDD) between T3 and T6 and previous thoracic SCS placement, with the tip positioned at the mid-T4 level.

**Figure 1:**
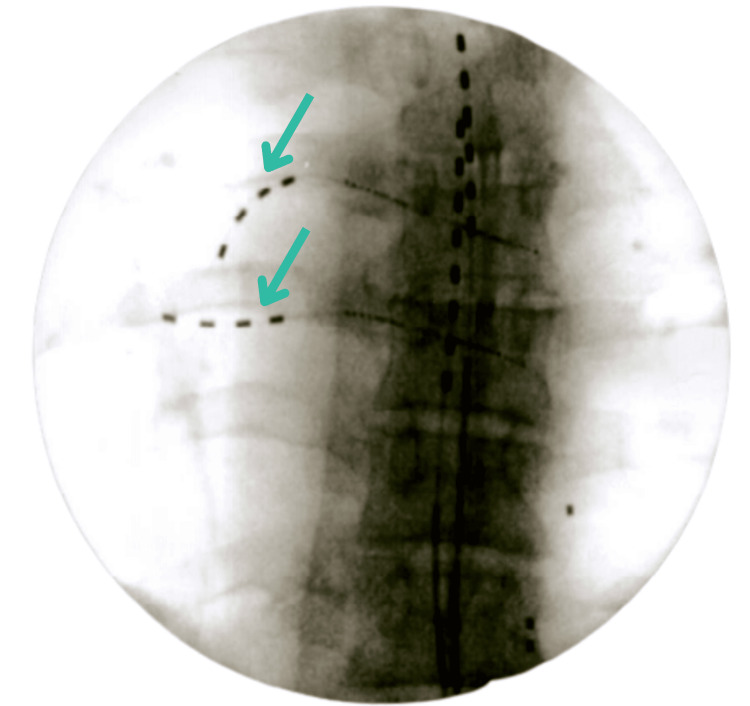
Anteroposterior view of T6 and T7 percutaneous peripheral nerve stimulator lead implants with pre-existing SCS thoracic leads. SCS: spinal cord stimulation

Given the patient’s extensive history of failed treatment regimens, a diagnostic and prognostic left intercostal nerve block was performed on the T6 and T7 nerves. Within two hours, the patient had significant post-procedure symptom relief, including a 70% reduction in pain. Due to the great response of the intercostal nerve block, the patient underwent implantation of a 60-day peripheral nerve stimulator (PNS) (SPRINT, SPR Therapeutics Inc., Cleveland, OH) for pain management of refractory intercostal neuralgia. The PNS trial stimulation parameters were set at 100 Hz for sensory stimulation on his left T6 and T7 nerves (Figure 1). 

At the 18-day follow-up, the patient's pain decreased from eight out of 10 to one out of 10 per the numeric rating scale (NRS). At the 52-day follow-up appointment, the patient continued to experience improvements in his neuropathic pain but continued to experience deep axial pain. To better manage the patient's residual symptoms, the left lead was replaced and targeted to the left medial branch nerve at the T7 level, leading to a 50% reduction in pain. Unfortunately, 18 days after the new lead aimed at the left T7 medial branch nerve was implanted, the patient developed superficial cellulitis, requiring an explant and treatment with cephalexin. Following the explant of the lead, the patient reported a return to his baseline level of pain. Due to the return of the patient’s pain, a permanent PNS system was discussed in detail, and the patient decided to proceed with this intervention.

**Figure 2:**
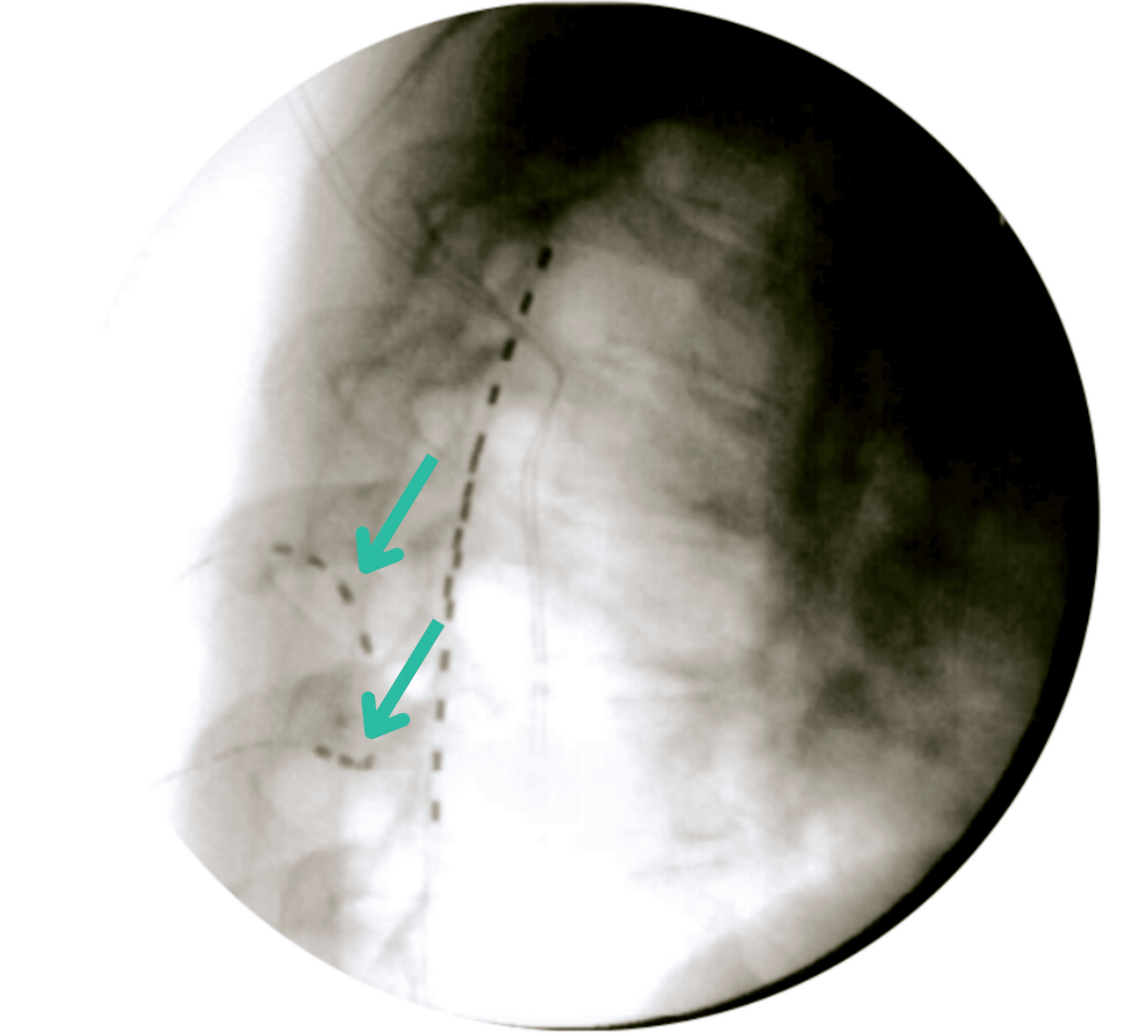
Lateral view of T6 and T7 peripheral nerve stimulator lead implant.

The patient initially underwent a PNS trial with the Curonix system (Curonix, Pompano Beach, FL), targeting the left T6 and T7 intercostal nerves (Figure 2). The patient reported a 50% pain reduction on day 10 and a 100% reduction on day 17. Due to the significant symptom relief the patient experienced with the PNS trial, the decision was made to proceed with the placement of the permanent PNS implant for long-term pain management. The permanent implant immediately improved 80% of the patient’s pain, which increased to 100% relief when system positioning was optimized. The settings were programmed at a pulse width of 60 μs, a frequency of 1 kHz (1000 Hz), and an intensity of 11.0 mA in each lead. Although the patient did experience some mild residual pain, it was well managed with the patient’s baseline 20 mg daily duloxetine. In this case, the patient had consistent and sustained relief of his intercostal neuralgia between 80-100% improvement at the one-, three-, and six-month follow-up.

At the two-year follow-up, the patient maintained significant pain relief with only occasional pain rated as a two out of 10. Fortunately, the patient was able to completely wean off opioid medications following the permanent implantation of the PNS and successfully returned to his daily activities, leading to an enhanced quality of life.

## Discussion

The exact mechanism of PNS is still unknown; however, a cornerstone of neuromodulation is the "gate control" theory of pain established by Melzack and Wall in 1960 [12]. This mechanism inspired the idea of using non-painful stimuli to large afferent Aβ fibers (e.g., those carrying pressure, touch, and vibration sensation) to inhibit the downstream transmission of nociceptive C pain fibers [12]. Some studies have highlighted the role of PNS in the modulation of higher central nervous system centers [13] and the effect of PNS on the different pathways of the spinal cord, including gabanergic, glycinergic, and NMDA pathways [14]. In this case, we describe the successful treatment of chronic neuropathic pain by PNS in a patient with a prolonged history of intractable intercostal neuralgia. 

PNS electrodes are often placed percutaneously near the targeted nerve via fluoroscopic guidance. However, some literature has demonstrated the feasibility of using ultrasound to facilitate the implantation of PNS leads [15]. By using ultrasound in conjunction with fluoroscopy, we leveraged the strengths of both modalities, thereby enhancing the precision and safety of the lead implantation process.

PNS devices allow customization of settings that allow modification of the waveform impulses to the nerve. Currently, there is limited evidence regarding the use of PNS with high frequencies (>1000 Hz), specifically for patients with neuropathies such as intercostal neuralgia. One clinical trial has demonstrated positive results by targeting multiple nerves with high-frequency stimulation using peripheral nerve stimulators, highlighting the potential effectiveness of this approach [16]. In addition, previous literature has explored the application of high-frequency stimulation, such as 10 kHz, in SCS and shown promising outcomes [16,17].

In our case presentation, high frequencies of 1000 Hz were used with the permanent PNS implant, as this demonstrated better relief for the patient. Although the patient had significant improvements in his symptoms using a 60-day PNS, they did not last after the 60-day trial. Ultimately, through shared decision-making, the patient elected for placement of a permanent nerve stimulator for ongoing relief. Upon placement of a permanent PNS system, the patient was able to get back to his daily activities, which were once limited due to chronic pain. The patient maintained long-standing relief of 80-100% at the one-year and two-year follow-up visits. This result indicates that PNS may successfully manage intercostal neuralgia pain in patients who have failed conservative measures.

The literature discussing the use of PNS in treating intercostal neuralgia is limited. Most reported cases involve individuals with post-herpetic neuralgia and multiple comorbidities [2-5,18]. However, long-term outcomes following traumatic causes of intercostal neuralgia treated with PNS have yet to be reported. Of the few cases that exist, there is only short-term follow-up that does not provide information on sustained relief for patients. To our knowledge, this two-year follow-up is the longest-reported outcome in the literature for the use of PNS for intercostal neuralgia after traumatic injury.

## Conclusions

The case presented suggests that PNS may effectively manage refractory chronic intercostal neuralgia and provide sustained relief to help patients return to their daily activities. PNS is a minimally invasive procedure that can be applied under ultrasound or fluoroscopic guidance to target specific pain-producing nerves. Efficacious treatment modalities, such as PNS, may lead to long-term symptom management and decrease the use of opioid medications for patients.
